# Hypofractionated stereotactic radiation therapy for recurrent glioblastoma: single institutional experience

**DOI:** 10.1186/1748-717X-8-222

**Published:** 2013-09-25

**Authors:** Patrizia Ciammella, Ala Podgornii, Maria Galeandro, Nunziata D’Abbiero, Anna Pisanello, Andrea Botti, Elisabetta Cagni, Mauro Iori, Cinzia Iotti

**Affiliations:** 1 Radiation Therapy Unit, Department of Oncology and Advanced Technology, Azienda Ospedaliera ASMN, Istituto di Ricovero e Cura a Carattere Scientifico, Viale Risorgimento 80, 42123 Reggio Emilia, Italy; 2 Neurology Unit, Dipartimento Neuro-Motorio, Azienda Ospedaliera ASMN, Istituto di Ricovero e Cura a Carattere Scientifico, Viale Risorgimento 80, 42123 Reggio Emilia, Italy; 3 Medical Physics Unit,Department of Oncology and Advanced Technology, Azienda Ospedaliera ASMN, Istituto di Ricovero e Cura a Carattere Scientifico, Viale Risorgimento 80, 42123 Reggio Emilia, Italy

**Keywords:** Recurrent glioblastoma, Stereotactic radiation therapy, Re-irradiation, Acute toxicity

## Abstract

**Background:**

Glioblastoma (GBM) is the most common malignant primary brain tumor in adults. Tumor control and survival have improved with the use of radiotherapy (RT) plus concomitant and adjuvant chemotherapy, but the prognosis remain poor. In most cases the recurrence occurs within 7–9 months after primary treatment. Currently, many approaches are available for the salvage treatment of patients with recurrent GBM, including resection, re-irradiation or systemic agents, but no standard of care exists.

**Methods:**

We analysed a cohort of patients with recurrent GBM treated with frame-less hypofractionated stereotactic radiation therapy with a total dose of 25 Gy in 5 fractions.

**Results:**

Of 91 consecutive patients with newly diagnosed GBM treated between 2007 and 2012 with conventional adjuvant chemo-radiation therapy, 15 underwent salvage RT at recurrence. The median time interval between primary RT and salvage RT was 10.8 months (range, 6–54 months). Overall, patients undergoing salvage RT showed a longer survival, with a median survival of 33 vs. 9.9 months (p= 0.00149). Median overall survival (OS) from salvage RT was 9.5 months. No patients demonstrated clinically significant acute morbidity, and all patients were able to complete the prescribed radiation therapy without interruption.

**Conclusion:**

Our results suggest that hypofractionated stereotactic radiation therapy is effective and safe in recurrent GBM. However, until prospective randomized trials will confirm these results, the decision for salvage treatment should remain individual and based on a multidisciplinary evaluation of each patient.

## Introduction

The current standard of care for glioblastoma (GBM) is concurrent Temozolomide (TMZ) and conventional radiotherapy followed by 6 maintenance cycles of adjuvant TMZ. Despite standard of care therapy, recurrence rates remain high (>90%) with a median overall survival (OS) of 15–18 months [1].

In recent years a lot of mono-therapy or combination chemotherapeutic strategies have been evaluated in patients with recurrent or progressive GBM. The alkylant agents (nitrosoureas, procarbazine), used in the past as first-line treatment, are used today as second-line therapy for recurrence or progression. Overall, the 6-months progression-free survival (PFS) and median OS with second-line therapy with alkylant agents ranged from 13 to 24% and from 5 to 11 months, respectively [2-8]. Numerous trials have evaluated the efficacy and safety of TMZ as a monotherapy for recurrent GBM in patients previously treated with chemotherapy, mostly nitrosurea-based [9-15]. Some recent studies evaluated in TMZ-pretreated patients a second-line TMZ based therapy [16-21]. In one of these studies, the TMZ resulted more efficacious than procarbazine (PFS at 6 months= 21% vs 8%) [16]. Six recent trials of TMZ-pretreated patients evaluated TMZ re-challenge [17-21]. Unfortunately, considering the small numbers of patients included in these studies and the wide range of TMZ regimen tested, there was no evidence that one regimen was advantageous over another, and despite some improvements in PFS none of them was associated with a better survival. The repeated surgery is feasible only in few cases of recurrent GBM and is not recommended for patients with involvement of pre-specified eloquent/critical brain regions [22]. The data related to reoperation have been derived from retrospective studies, but it is known that positive prognostic factors of reoperation for recurrent GBM are a younger age (≤70 years), a smaller tumor volume (≤50 cm3) and pre-operative KPS greater than 80% [22,23].

Today, for recurrent GBM the re-irradiation remains a palliative option for a selected group of patients, i.e. those with a KPS greater than 60%, a tumor size of up to 40 mm, and a time to progression from the surgery of at least 6 months [24]. Usually, a stereotactic radiotherapy technique is applied [25], but the fractionation schedule and the prescribed total dose are various and no randomized trials are available. Both single-fraction stereotactic radiosurgery (SRS) and fractionated stereotactic radiotherapy (FSRT) have been reported to have minimal toxicity, with median survivals of 6–10 months after SRS [26-30] or FSRT [31-33]. Recently, some studies also have evaluated the safety and efficacy of the combination of fractionated RT and concurrent chemotherapy in recurrent high-grade gliomas showing a median OS and PFS from re-irradiation of 8–13 and 5–8 months, respectively [34-37].

Our retrospective analysis focused on the efficacy and toxicity of a hypofractionated stereotactic radiotherapy for recurrent GBM in a cohort of patients previously treated with standard therapy.

## Methods and materials

This is a retrospective study of efficacy and safety of hypofractionated stereotactic radiation therapy (SRT) delivered at 5 fractions of 5 Gy each for management of recurrent intracranial GBM. All patients previously underwent a conventional adjuvant chemo-radiation therapy according to Stupp protocol after a maximal surgical resection for a newly diagnosed GBM. The histopathological diagnosis of primary GBM was established according to the current World Health Organization Criteria [38].

The protocol was approved by the local Ethics committee and informed consent was obtained for all patients.

### Recurrence definition

The diagnosis of tumor recurrence was based on the joint opinion of the neuro-radiologist, neurosurgeon, radiation oncologist and neuro-oncologist, and was defined as appearance of new contrast-enhanced lesion(s) on T1-weichted MRI or an increase of 25% or more of the volume of the initial enhanced lesion(s). In case of doubt between tumor recurrence and pseudo-progression a MR imaging was repeated after one month. Recurrences were defined, as suggested by Lee et at [39], as " in-field" if > 80% of the tumour recurrence resided within the prescription 95% isodose surface, and "marginal" if 20% to 80% of the lesion was inside the 95% isodose surface. In all other cases, recurrences were defined as outside of the radiation field. At the time of recurrence, all patients were evaluated for salvage treatment, which included re-resection of the tumor, hypo-fractionated radiation therapy, chemotherapy, or combined approaches.

### Re-irradiation protocol

The indication for re-irradiation at tumor recurrence was evaluated by the interdisciplinary neuro-oncology team and based on: the patient clinical condition (i.e. KPS> 60), the lesion location and the spread of disease (patients with multifocal spread of disease were excluded). All patients undergoing re-irradiation were immobilized in customized thermoplastic shells for CT and MR simulation. The GTV was delineated on the basis of the contrast-enhancing tumor on T1-weighted MRI registered with CT images. For the image registration an automatic fusion algorithm was used; anyway the resulted registration was subsequently checked by mean of anatomic markers and, if needed, manually adjusted. The PTV was generated by applying to GTV an isotropic expansion of 3–5 mm. The critical structures and previously high dose irradiated volumes were contoured. All patients were treated with hypo-fractionated stereotactic radiation therapy with multiple no-coplanar beams using on a standard 6-MV linear accelerator (LINAC). A total dose of 25 Gy prescribed to the 70% isodose line and delivered in 5 consecutive fractions was delivered. A daily pre-treatment verification with orthogonal fields was performed.

### Follow-up protocol

All patients were evaluated at four weeks, 12 weeks and once every 3 months after the salvage treatment. The work-up included history and physical examination (with particular regard to KPS, neurological status and toxicity assessment), blood tests and contrast-enhanced brain MRI. No specific quality-of-life questionnaire was administered. Haematologic and no-haematological toxicities were graded according to Common Terminology Criteria for Adverse Events. Other investigations were not performed unless were clinically indicated.

### Statistical analysis

Statistical analysis were performed using the R statistical software (http://www.R-project.org), particularly the survival package. Patient characteristics were compared with Fisher-test and a twosided significance level was chosen at 0.05. Survival curves were estimated using the Kaplan-Meier method. The p-values estimated are those for a two tailed test and the significance level was chosen to be 5%. Cox regression analysis was used for univariate analysis and parameters were tested for proportionally and categorical covariates compared by log-rank test. A multivariate Cox proportional hazards analysis was used to evaluate prognostic factors and treatment with respect to the overall survival from initial diagnosis and from time of recurrence. Variables included in the model were selected when statistically significant in univariate analysis. Data are presented as 1-mont actuarial hazard ratios (HR) with 95% confidence intervals, unless otherwise mentioned.

## Results

Between 2007 and 2012 we treated with standard chemo-radiation therapy according to Stupp protocol 91 patients with newly diagnosed GBM. With mean follow-up of 13.9 months (range 1–63 months), the median OS was 15 months, with a PFS of 9 months. In this populations of patients, the age (≤65 years), extent of resection, KPS, RPA class and O6-methylguanine-DNA-methyltransferase (MGMT) methylation were significantly associated with OS.

The pattern of recurrences was analysed in 83 patients who had recurrences. Recurrence occurred "in-field" in 61 patients (73%), at RT field margin in 7 patients (9%) and "out-field" in 15 patients (18%). No patients had CSF/spinal or distant disease recurrence. No correlation between extent of surgery and site of relapse was seen (>0.05). Progression-free survival did not differ significantly between patients with regional or marginal progression and patients with distant recurrences. Comparison of the investigated clinical factors did not differ significantly between the two groups ("in-field" vs "out-field"). No correlation between tumor location, tumor side, extent of primary surgery and pattern of recurrence was found.

**Figure 1:**
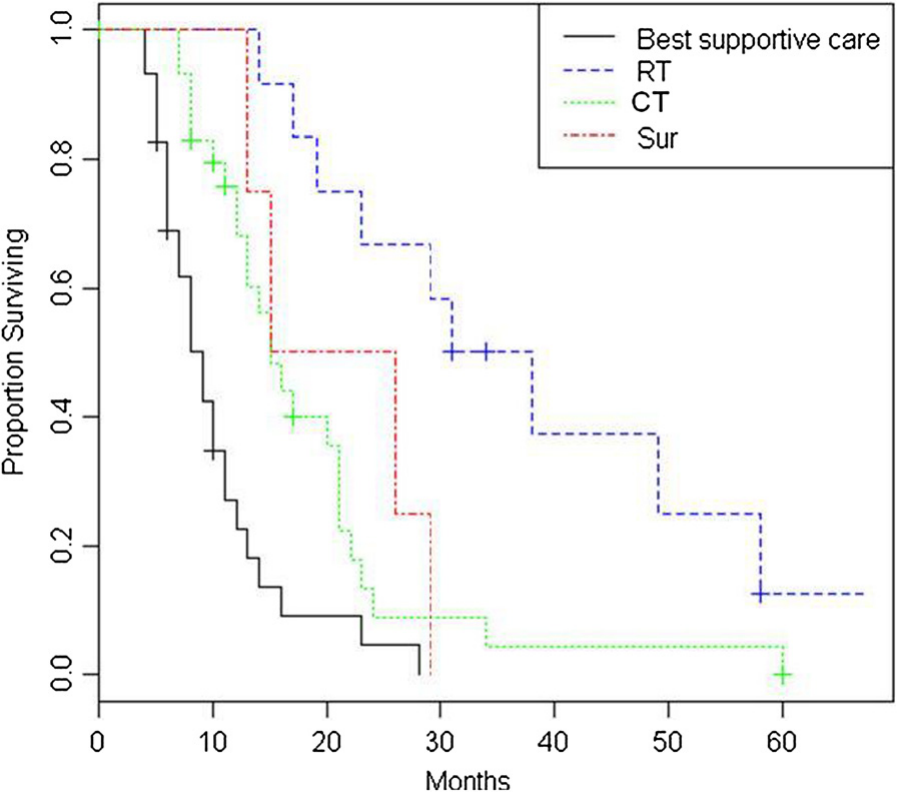
Kaplan-Meier analysis of overall survival from the time of initial diagnosis in patients with recurrent GBM treated with radiotherapy (RT), surgery (Sur), chemotherapy (CT) or best supportive care.

**Figure 2:**
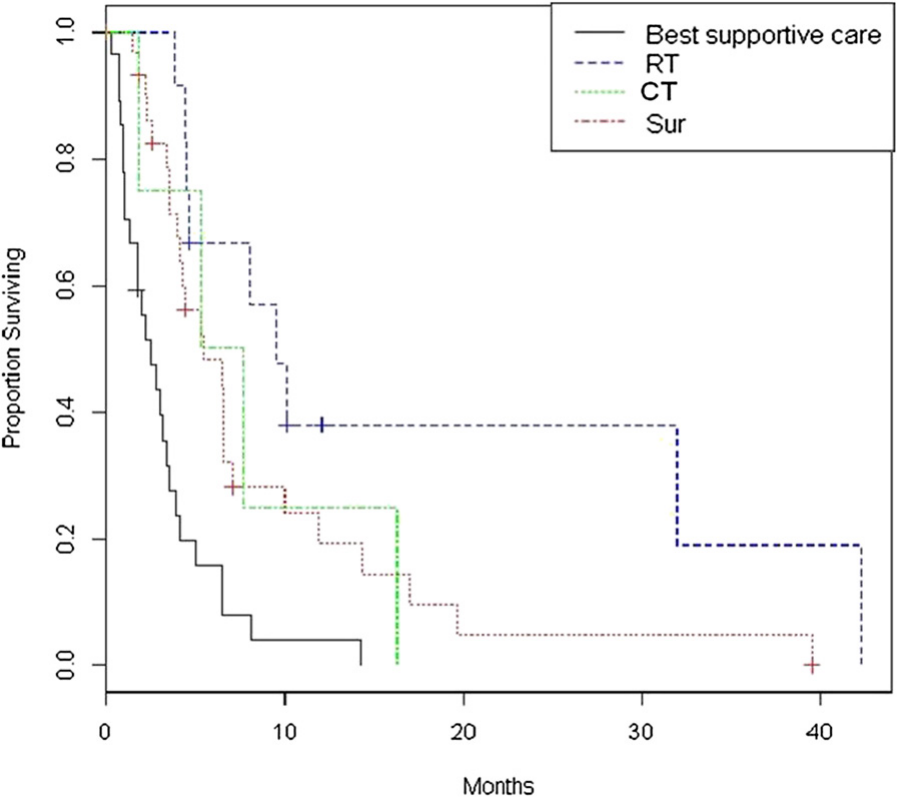
Kaplan-Meier analysis of overall survival from the time of recurrence in patients with recurrent GBM treated with radiotherapy (RT), surgery (Sur), chemotherapy (CT) or best supportive care.

At the time of recurrence, re-surgery was performed in 6 patients and second line chemotherapy in 37 patients. The salvage re-irradiation was performed in 9 of 58 patients with regional or marginal tumor progression and in 6 of 13 patients with distant recurrence. The remaining patients with tumor recurrence not eligible for salvage therapies (due to old age, poor performance status, severe comorbidities, patient choice) have received best supportive care. Table 1 shows the comparison between the different types of approaches for recurrent GBM (re-irradiation, re-surgery, second-line chemotherapy and best supportive care) in terms of patients’ characteristics and clinical outcomes. The median time interval between primary RT and re-irradiation was 10.8 months (range, 6–54 months). No patients demonstrated clinically significant acute morbidity, and all patients were able to complete the prescribed treatment without interruption. No patient required hospitalization or surgery for early acute or delayed toxicity. Neurological deterioration occurred in two patients at 1 and 3 months after re-irradiation and was managed successfully with dexamethasone. Log-rank test revealed that the patients undergoing re-irradiation showed a longer survival compared to those treated with best supportive care, with a median overall survival from primary GBM diagnosis of 33 vs 9.9 months (p= 0.0015). As well the patients treated with re-surgery or second line chemotherapy showed a longer survival than untreated patients, with a median OS from diagnosis of 17 months. The median time of recurrence from primary GBM diagnosis was statistically significant higher in the re-irradiation group when compared to all patients (19 months, 10 months, 8 months and 5 months in RT, surgery, chemotherapy and best supportive care, respectively, p= 0.00003). This finding may explain the higher overall survival of patients undergoing re-irradiation. The median survival from recurrence was 9.5 months, 5.5 months and 2.5 months for RT, chemotherapy and best supportive care group, respectively. This difference was statistically significant between RT and best supportive care group (p= 0.000001), between chemotherapy and best supportive care group (p= 0.00007) and between RT and second-line chemotherapy group (p=0.049). The Figures 1 and 2 show the Kaplan-Meier curves for OS from primary GBM diagnosis and from recurrence, comparing those treated with RT, surgery, chemotherapy and best supportive care.

**Table 1:** Comparison of clinical factors and outcome variables in patients with recurrent glioblastoma treated with different approaches

	**Salvage treatment**				
**Characteristics**	**Re-irradiation**	**Re-surgery**	**Chemotherapy**	**Best supportive care**	***p Value***
	**(N= 15)**	**(N=6 )**	**(N=37 )**	**(N=25 )**	
Age (years)					
Median	51.5	65	66	66	RT vs no-RT 0.002
Range	41-73	55-73	49-72	55-79	
<60 years	5	3	21	20	
<=60 years	10	3	16	5	0.02
Gender, N0. (%)					
Men	11 (73%)	4 (67%)	22 (59%)	9 (38%)	0.33
Women	4 (27%)	2 (33%)	15 (41%)	16 (62%)	
Primary Surgery, N0. (%)					
Total	11 (73%)	4 (67%)	18 (49%)	10 (39%)	0.18
Subtotal	3 (20%)	2 (33%)	11 (30%)	4 (15%)	
Biopsy	1 (7%)	0	8 (21%)	11 (46%)	
Karnofsky performance status at diagnosis (%)					.
Median	90	90	90	90	n.s
Range	80-100	80-100	70-100	70-100	
RPA classification at diagnosis, N0. (%)					
IV	9 (60%)	5 (83%)	18 (50%)	13 (54%)	
V	5 (33%)	1 (17%)	8 (20%)	6 (23%)	
VI	1 (7%)	0	11 (30%)	6 (23%)	
MGMT Methylation, N0. (%)					
Yes	7 (47%)	4 (67%)	12 (33%)	10 (38%)	0.47
No	5 (33%)	2 (33%)	20 (54%)	13 (54%)	
Unknown	3 (20%)	0	5 (13%)	2 (8%)	
Primary Tumor location, N0 (%)					
Frontal	3 (20%)	3 (50%)	12 (34%)	10 (39%)	0.72
Temporal	4 (27%)	3 (50%)	13 (35%)	6 (23%)	
Parietal	6 (40%)	0	8 (21%)	6 (23%)	
Occipital	2 (13%)	0	4 (10%)	3 (15%)	
Primary Tumor side, N0 (%)					
Left	8 (53%)	4 (67%)	20 (54%)	14 (54%)	0.96
Right	7 (47%)	2 (33%)	17 (46%)	11 (46%)	
Type of recurrence, N0 (%)					
In-field	9 (60%)	5 (83%)	32 (86%)	19 (77%)	
Out-field	6 (40%)	1 (17%)	5 (14%)	6 (23%)	0.19
Karnofsky performance status at recurrence (%)					
Median	90	90	80	80	n.s.
Range	80-100	80-100	70-100	70-100	
Median OS from primary diagnosis (months)	33	17	17	9.9	RT vs no-RT 0.001
*Cox regression analysis OR coef f-p*	0.102	0.268	0.331	1	
	1.4e-07	0.016	0.00018	-	
Time between primary therapy and salvage treatment					
Median (months)	10.8	10	8	5	0.003
Range (months)	6-54	7-24	7-21	2-11	
Median OS from recurrence(months)	9.5	5.5	5.5	2.5	RT vs no-RT 0.001
*Cox regression analysisOR coef f-p*	0.16	0.34	0.33	1	
	0.000016	0.052	0.0015	-	

The clinical characteristics of the patients in the four treatment groups were similar, except for the median age that was significantly lower in the RT group (p<0.05). The patients who underwent re-irradiation and re-surgery showed better clinical conditions (KPS) compared with patients treated with second-line chemotherapy or untreated (best supportive care), but the difference did not reach statistical significance.

In all re-treated patients, the multivariate Cox proportional hazard analysis confirmed the negative prognostic effect of the older age [hazard ratio (HR): 4.1 (95%CI: 1.5 -10.3), p=0.005] and the biopsy alone [HR: 4.5 (95%CI: 1.4–14.4), p= 0.011] on the OS. No correlation was found between the other analysed factors (sex, MGMT methylation, RPA class, primary tumor side, primary tumor volume, KPS at diagnosis and KPS at recurrence) and OS. In all recurrent patients there was no correlation between OS from recurrence and type of recurrence ("in-field" or "out -field"), though in the group of re-irradiated patients with "in-field" progression had a mild tendency to better OS comparing with patients with "out-field". Surprisingly, in the re-irradiated group, the age alone (with cut-off of 60 years) showed a correlation with OS [HR: 11.92 (95%CI: 1.1-135), p=0.0045]. None of the others assessed factors reached statistical significance.

## Discussion

This retrospective study reports the results of a series of patients affected by GBM treated with conventional chemo-radiation therapy ad diagnosis and who underwent various salvage treatment for their tumor recurrence. The principal focus of our analysis is to evaluate the feasibility and safety of re-irradiation at tumor progression in patients previously irradiated. It is well known that the tumor control and survival in patients with GBM have been improved by the use of radiotherapy plus concomitant and adjuvant TMZ. Unfortunately, the prognosis remain poor with very few long-term survivors. In most cases the recurrence occurs within 7–9 months from initial diagnosis [40-42]. Up to 90% of all GBMs relapse in close proximity to the resection cavity or to the target volume of postoperative radiotherapy [43,44]. Currently, many approaches are available for the salvage treatment of patients with recurrent GBM after initial chemo-radiation therapy, including resection, re-irradiation or systemic agents, but no standard of care exists. Chemotherapy is the most common treatment option for recurrent GBM, but it is associated, when administered alone, with poor OS ( 4–6 months) [41].

The drugs commonly used in the treatment of these patients are many (TMZ, carboplatin, procarbazine, bevacizumab and imatinib) administered as single-agent or in combination. Recent series reported promising results with TMZ alone with a 6 months PFS of 30% [13]. The use of Bevacizumab was approved by the US Food and Drug Administration because of some phase II trials indicating prolonged 6-month PFS and OS [45]. Several studies suggest that a more aggressive approach with gross total resection and/or high dose re-irradiation may result in improvement of local control of recurrent GBM. A recent review reported an OS of 3–11 months in patients with recurrent GBM treated with surgery [23]. Young et al. [46] reported in 24 patients with tumor recurrence treated with surgery alone an OS of 3.3 months. With a combination of re-intervention and chemotherapy, an increase of PFS and OS was reported [47].

During the last decade, there has been an increased interest in fractionated stereotactic radiation therapy as a palliative treatment of recurrent GBM.

Combs et al. reported a median OS after re-irradiation of 8 months by applying normo-fractionated stereotactic radiotherapy [32]. The same authors have found an OS of 10 months in patients undergoing single-fraction stereotactic radiosurgery for recurrent GBM [28]. Vordermark et al. [33] showed a median OS of 7.9 months after hypo-fractionated stereotactic radiation therapy in 19 patients with recurrent GBM. Sirin et al. reported an overall survival from recurrence of 9.3 months after radiosurgery for recurrent GBM [48]. Similar results have been reported by other studies summarized in Table 2[30-34,49-63]. But a comparison of clinical outcomes provided by the application of so many and different approaches of re-irradiation certainly remains difficult because of the variation in target definition, treatment technique, total dose and dose/fractions, in the introduction of concomitant chemotherapy and initial patient characteristics.

**Table 2:** Survey of clinical outcomes after re-irradiation: fractionated stereotactic radiation therapy (FSRT), radiosurgery (SRS), brachytherapy (BT) of recurrent GBM

**Authors**	**Patients (n)**	**Type of RT**	**Total dose/fractions**	**Outcomes from the re-irradiation**
Cho *et al.*[30]	25	FSRT	Median dose of 37.5 Gy (range, 20-45 Gy) /2.5 Gy fractions (range, 1.8-3 Gy)	Median survival 12 months
Cho *et al.*[30]	46	SRS	Median total dose of 17 Gy delivered to the median of 50% isodose surface	Median survival 11 months
Combs *et al.*[31]	59	FSRT	36 Gy/2 Gy fractions	Median OS 8 months
				1-year survival rates 23%
				Median PFS 5 months
				1-year PFS 5%
Vordermark *et al.*[33]	19	FSRT	Median total dose 30 Gy (range, 20–30 Gy) /5 Gy fractions (range, 4–10 Gy)	Median OS 7.9 months
Simon *et al.*[49]	42	Iridium BT	50 Gy	Median OS 12.5 months
Chan *et al.*[50]	24	BT	53 Gy	Median OS 9.1 months
Larson *et al.*[51]	14	SRS	15 Gy/1 fraction	Median OS 9.5 months
Combs *et al.*[32]	32	SRS	15 Gy/1 fraction	Median OS 10 months
Shrieve *et al.*[52]	86	SRS	13 Gy/1 fraction	Median OS 10.2 months
Shrieve *et al.*[52]	32	BT	50 Gy	Median OS 11.5 months
Grosu *et al.*[53]	33	FSRT	30 Gy	Median OS 8 months (for astrocytomas and gliomas)
Kohshi *et al.*[54]	25	FSRT	22 Gy	Median OS 11 months
Ernst-Stecken *et al.*[55]	15	FSRT	35 Gy/7 Gy fractions	6 months PFS 75%
				12 months PFS 53%
Fokas *et al.*[56]	53	FSRT	Median dose 30 Gy (range 20-60 Gy)/ 3 Gy fractions (range 2-5 Gy)	Median OS 9 months
				1-year PFS 22%
				2-year PFS 5%
Henke *et al.*[57]	31 (2 grade III, 29 grade IV)	FSRT	Median total dose 20Gy (range, 20–25)/ 5 Gy fractions	Median OS 10.2 months,
Fogh *et al.*[58]	147 (42 grade III, 105 grade IV)	FSRT	Median dose 35 Gy in 3.5-Gy fractions	Median OS 11 months for grade III and 8 months for grade IV
Shepherd *et al.*[59]	29	FSRT	Median dose 35 Gy (range, 20–50 Gy)/ 5 Gy fractions	Median OS 10.7 months
Glass *et al.*[60]	20 (7 grade III, 13 grade IV)	FSRT	Median dose 38 Gy (range, 35–42 Gy)/ 3.5–6 Gy fractions	Median OS 12.7 months
Hudes *et al*. [61]	19	FSRT	Median dose 30 Gy (range, 24–35 Gy)/ 3–3.5 Gy fractions	Median OS 10.5 months
Lederman *et al.*[34]	88	FSRT	Total dose 18–36/ 4–9 Gy (weekly)	Median OS 7 months
Voynov *et al.*[62]	10 (5 WHO grade III, 5 grade IV)	FSRT	30 Gy /5 Gy fractions	Median OS 10.1 months

A recent review has analysed data from more than 300 GBM patients and has demonstrated that re-irradiation yields an increase of the 6-month PFS, that moves from 28% to 39% and 1-year overall survival of 18% to 48%, without additional chemotherapy [64]. A clinical improvement was observed in 24% to 45% of the cases with patients with KPS <70 apparently having lesser benefit from re-irradiation. Recently particular attention received the combination of stereotactic radiation therapy and TMZ in recurrent GBM. The use of TMZ in addition to radiotherapy was based on the observation that concurrent chemotherapy can potentiate the cytotoxicity of radiation. In a series of 25 patients with recurrent GBM treated at dose of 36 Gy in 2-Gy fractions in combination with TMZ, the reported median OS and PFS from re-irradiation were 8 and 5 months, respectively [36]. Grosu et al. reported a median survival time of 11 months for patients who have received fractionated stereotactic radiation therapy plus TMZ, while a survival time of 6 months was reached by patients treated with fractionated stereotactic radiation therapy without TMZ (p=0.0008) [53]. Literature is sparse regarding the toxicity of hypo-fractionated stereotactic radiotherapy in recurrent GBM. Some studies reported higher rates of necrosis but because of the wide range of delivered doses the data are inconclusive. However, the comparison of different studies of re-irradiation remains difficult because of the variability in target definition, treatment technique, fractionation schema, use of concomitant chemotherapy and initial patient characteristics. In the management of GB, the pattern of failure is one of the major concerns in relation to the clinical target volume margins, optimal radiation dose, and identification of biomarkers. In the majority of reported series, local progression after initial management was encountered in 60% to 97% of cases, but comparison of different studies is difficult due to differences in treatment strategy, extension of surgical removal, postoperative surveillance, length of follow up as well as definition and categorization of the tumor progression. Several studies have shown that the majority of patients with GBM treated with RT plus concomitant and adjuvant temozolomide have central recurrences [65-68]. Furthermore, many previous trials found that the type of local recurrence in relation to the radiation fields ("in field", "marginal" or "out-field" recurrence) was associated with impaired prognosis [68-73]. In particular, the median survival was 17.3, 14.8 and 26.1 months in patients with recurrence inside, at the margin and outside the irradiation field [71].

The objective of the current study is to evaluate retrospectively the efficacy and the safety of the re-irradiation of patients with recurrent GBM in comparison with other salvage approaches. The patients of the study were part of a consecutive series of 91 patients previously treated with standard RT and TMZ.

In our series the pattern of recurrences evaluated in 71 cases was : "in-field" recurrence in 51 patients (73%), marginal recurrence in 7 patients (9%) and "out-of-field" recurrence in 13 patients (17%). At tumor recurrence/progression, only 15 and 6 patients received a salvage radiation treatment or a re-operation, respectively. The remaining 50 patients with disease progression had been considered un-fit for local therapy and received a second line chemotherapy (37 patients) or the best supportive care (13 patients). The median survival time after diagnosis of tumor recurrence in the group of patients treated with re-irradiation (9.5 months) was significantly longer than that who received best supportive care (2.5 months, p=0.0001) and which has had a longer trend compared to that of patients treated with chemotherapy of second line(5.5 months, p=0.049). Furthermore, we have found that patients treated with re-irradiation at the time of tumor recurrence has had the longest overall survival from the time of initial diagnosis (33 months, p=0.000049). However, we can not exclude that this finding may result from selection bias for application of the different types of salvage treatment. Also the patients who underwent re-surgery show to survive less than re-irradiated patients (median OS 33 vs 17 months respectively, p= 0.00034). We did not found a significant correlation between the OS and the pattern of failure, although in the group of patients with "in-field" rather than outside progression a mild tendency to a better OS was observed.. This trend might reflect an other selection bias, due to the possible inclusion of some cases of pseudo-progression erroneously interpreted as local recurrence.

With regard to the time interval between the first to the second irradiation, we found a positive but not significant correlation between longer interval and survival after re-irradiation.

Unfortunately, our study represents a retrospective analysis and patients at the time of tumor recurrence were not randomized to one or the other salvage treatment. In addition, the indication for the salvage treatment and the choice of which perform (surgery, re-irradiation, chemotherapy) was evaluated by our interdisciplinary neuro-oncology team (and from the patient preferences too). The clinical and tumoral features that have guided their decision were mainly: the patient clinical condition (i.e. KPS> 70), the lesion location and the spread of disease. This means that patients with up-front worse prognosis were excluded from the re-irradiation. Stratifying for these characteristics, the analysis of all patients showed no differences between the groups of different salvage strategies and did not allow the identification of factors associated with choice of one or other salvage treatment. On the basis of these considerations, it was not possible to match the patients included in these groups and therefore the drawing of any conclusions regarding the efficacy of each treatment modality it can suffer of some bias and/or arbitrary.

On the other hand, our results confirm the feasibility and safety of re-irradiation in recurrent GBM. In fact, in our population, although it is a small series, re-irradiation was not accompanied by any case of significant morbidity or side effect. Literature is sparse regarding the toxicity of hypo-fractionated stereotactic radiotherapy in recurrent GBM. Previous studies reported higher rates of necrosis but have utilized a wide range of doses. An association has been noted between higher rates of re-operation and doses greater than 40 Gy [59,74]. With the limitations of the data now at our disposal, radiation therapy for recurrent GBM after standard therapy seems to be safe and in our study the re-irradiated patients have a longer OS compared to patients treated with other approaches.

## Conclusion

Our study shows that hypo-fractionated stereotactic radiation therapy is effective and safe in recurrent GBM after conventional chemo-radiation treatment, even if the dose response and dose limits remain unclear. Anyway, until prospective randomized trials will confirm these results, the decisions for salvage re-irradiation should be based on multidisciplinary evaluation and personalized on the patient.

## Competing interests

All authors disclose no actual or potential conflict of interest including any financial, personal or other relationships with other people or organizations that could inappropriately influence their work.

## Authors’ contribution

PC, AP and CI participated in the design of the study. PC, AP, MG, AP and ND carried out the data and participated in the data evaluation. PC, AB, AP and EC performed the statistical analysis. PC and AP drafted the manuscript. The definitive supervision of the paper was done by CI and MI. All authors read and approved the final manuscript.
